# Long-term efficacy of deep brain stimulation in *PLA2G6*-related Parkinson’s disease: A case report with literature review

**DOI:** 10.1016/j.prdoa.2025.100377

**Published:** 2025-07-28

**Authors:** Takashi Tsuboi, Takashi Uematsu, Yoshiki Ito, Tomotaka Ishizaki, Satoshi Maesawa, Ryuta Saito, Hiroyo Yoshino, Nobutaka Hattori, Adolfo Ramirez-Zamora, Michael S. Okun, Masahisa Katsuno

**Affiliations:** a Department of Neurology, Nagoya University Graduate School of Medicine, Japan; b Department of Neurosurgery, Nagoya University Graduate School of Medicine, Japan; c Department of Neurosurgery, National Hospital Organization, Nagoya Medical Center, Japan; d Research Institute for Diseases of Old Age, Graduate School of Medicine, Juntendo University, Tokyo, Japan; e Department of Neurology, Juntendo University School of Medicine, Tokyo, Japan; f Department of Neurology, Norman Fixel Institute for Neurological Diseases, University of Florida Health, United States

**Keywords:** *PLA2G6*, Parkinson’s disease, Dystonia, Deep brain stimulation, Globus pallidus internus

## Abstract

A patient with early-onset PARK14 and novel *PLA2G6* variants underwent globus pallidus internus deep brain stimulation, achieving sustained three-year motor and quality-of-life improvements. Although a literature review supports motor benefits, our case’s cognitive decline highlights the need for comprehensive assessment of non-motor symptoms and quality-of-life in this rare disorder.

*PLA2G6*-associated neurodegeneration (PLAN) is a rare autosomal recessive neurodegenerative disorder often associated with brain iron accumulation [1]. The clinical spectrum of PLAN includes infantile neuroaxonal dystrophy (INAD), atypical neuroaxonal dystrophy (ANAD) and adult-onset parkinsonism (PARK14). PARK14 typically presents as early-onset Parkinson’s disease (PD), often complicated by dystonia, pyramidal signs, and neuropsychiatric symptoms [2]. While levodopa offers initial benefit, patients frequently develop early and refractory motor complications, necessitating advanced therapies like deep brain stimulation (DBS) [[2], [3], [4], [5]]. Here, we present a case of PARK14 with novel compound heterozygous *PLA2G6* variants who underwent globus pallidus internus (GPi) DBS, followed by a literature review to contextualize the role of DBS in this disorder.

A 26-year-old man presented with lower extremity stiffness and an abnormal gait, with a background of depression, generalized anxiety, and frequent panic attacks. His paternal grandmother had a history of PD. At age 27, he developed a left-hand tremor, prompting referral to a movement disorder specialist. Neurological examination revealed resting and postural tremor in both upper limbs, with moderate rigidity and bradykinesia affecting all four limbs. During walking, he exhibited rightward trunk deviation with each left foot strike in a characteristic, reproducible pattern. The deviation disappeared during long-stride or backward walking, consistent with truncal dystonia (Video 1). Brain MRI showed minimal iron deposition not reaching pathological levels and no focal atrophy (Supplementary Fig. 1A). Dopamine transporter imaging demonstrated bilaterally reduced uptake, with specific binding ratios of 3.89/3.94 (right/left) and corresponding Z-scores of –4.04/–4.00 (Supplementary Fig. 1B). However, ^123^I-metaiodobenzylguanidine (MIBG) myocardial scintigraphy was normal, arguing against idiopathic PD (Supplementary Fig. 1C). Cognitive assessments were within the normal range: Mini-Mental State Examination (MMSE) 30 and Montreal Cognitive Assessment (MoCA) 27. He was diagnosed with early-onset PD.

Levodopa (up to 450 mg/day) improved parkinsonism but not dystonia. Trihexyphenidyl was added and titrated to 6 mg/day, resulting in mild dystonia improvement. The addition of zonisamide 50 mg/day further reduced tremor and parkinsonism. Seven months later, he was hospitalized for a suicide attempt. Subsequent genetic testing identified novel compound heterozygous *PLA2G6* variants (c.1977C>G (p.N659K) and c.2129G>A (p.R710H)), establishing a diagnosis of PARK14. Both variants were rare in public databases and predicted to be deleterious by multiple in silico tools. Details of allele frequencies and prediction scores are summarized in Supplementary Table 1.

Within 23 months of treatment, he developed severe, debilitating dyskinesias, leading to feeding difficulties, weight loss, and another hospitalization. OFF periods occupied 25 % of his day. Due to these refractory motor complications and his psychiatric history, bilateral GPi DBS was chosen over the subthalamic nucleus (STN) to minimize cognitive and psychiatric risks. Directional leads and a pulse generator (Vercise Cartesia and Vercise Genus DBS system, Boston Scientific, MA, USA) were implanted. Stimulation was initiated bilaterally: Case+, contacts 2/3/4- (33 %), 60 μs, 130 Hz, 1.3 mA. Dyskinesia and truncal dystonia were nearly completely controlled, and OFF periods were reduced (Video 1). OFF time was further reduced by increasing levodopa to 600 mg/day and adding opicapone 25 mg (equivalent to 50 mg capsule) and rasagiline 1 mg. Given dystonia improvement following DBS and the potential for cognitive side effects, trihexyphenidyl was discontinued.

At 3 years postoperatively, stimulation settings were 3.5 mA, 60 μs, and 130 Hz bilaterally. Motor function (Movement Disorders Society-Unified Parkinson’s Disease Rating Scale, MDS-UPDRS III) remained stable, motor complications (MDS-UPDRS IV) improved, and his quality of life (PDQ-39) was markedly enhanced (Table 1). However, his cognition declined (MMSE 26, MoCA 20), likely reflecting disease progression.

**Table 1:** DBS outcomes.

	Baseline	3 years
MDS-UPDRS part III (Medication-on state) a	26	20
Tremor subscore b	1	1
Rigidity subscore b	6	1
Bradykinesia subscore b	16	15
Gait and postural stability subscore b	2	1
MDS-UPDRS part III (Medication-off state)	34	NA
Tremor subscore b	1	NA
Rigidity subscore b	8	NA
Bradykinesia subscore b	21	NA
Gait and postural stability subscore b	3	NA
MDS-UPDRS part IV	12	6
Dyskinesia score c	7	0
PDQ-39 summary index	53	24
PDQ-39 mobility score	95	60
PDQ-39 ADL score	38	33
PDQ-39 emotional well-being score	75	54
PDQ-39 stigma score	56	0
PDQ-39 social support score	42	0
PDQ-39 cognition score	44	38
PDQ-39 communication score	17	0
PDQ-39 bodily discomfort score	58	8
SCOPA AUT	9	7
LEDD d	850	1100
MMSE e	30	26
MoCA e	27	21

Abbreviations: LEDD, Levodopa equivalent daily dose; MDS-UPDRS, Movement Disorder Society Unified Parkinson’s Disease Rating Scale; MMSE, Mini-Mental State Examination; MoCA, Montreal Cognitive Assessment; NA, not assessed; PDQ-39, Parkinson’s Disease Questionnaire-39; SCOPA AUT, Scales for Outcomes in Parkinson’s disease-autonomic dysfunction.

a The evaluation during the medication-on state was conducted approximately one hour after levodopa administration. Motor function at 3 years was assessed in the medication-on/stimulation-on state.

b Motor subscores were defined as follows: rigidity (MDS-UPDRS item 3.3); tremor (items 3.15–3.18); bradykinesia (items 3.2, 3.4–3.8, and 3.14); and gait/postural stability (items 3.9–3.13).

c Dyskinesia scores were derived from MDS-UPDRS items 4.1 and 4.2.

d Daily oral medications were levodopa 450 mg, trihexyphenidyl 6 mg, and zonisamide 50 mg preoperatively, and levodopa 600 mg, opicapone 25 mg, rasagiline 1 mg, and zonisamide 50 mg postoperatively.

e Cognitive assessments were performed in the medication-on state at baseline, and in the medication-on/stimulation-on state at 3 years.

To contextualize our findings, we conducted a literature review (PubMed, 2000–2025) and identified 10 previously reported PLAN patients treated with DBS: nine with the PARK14 phenotype and one with ANAD [[2], [3], [4], [5], [6], [7], [8], [9]]. Methodological details are provided in the Supplementary Material. The clinical features of these 10 cases, together with the present case, are summarized in Table 2. The patients were geographically diverse and varied in genotypes and clinical phenotypes, reflecting the heterogeneity of PLAN. PARK14 patients typically developed parkinsonism in early adulthood (17 to 39 years, median: 28 years), often accompanied by dystonia, pyramidal signs, cerebellar features, or myoclonus. Non-motor symptoms such as depression, anxiety, and cognitive impairment were frequently reported. Brain MRI was unremarkable in half of the cases, while others showed mild cerebellar atrophy or iron accumulation. Consistent with previous reports linking missense variants to milder phenotypes, most patients carried homozygous or compound heterozygous missense mutations [1].

**Table 2:** Clinical characteristics and DBS outcomes of our case and previously-reported cases.

Case	Our case	Case 1	Case 2	Case 3	Case 4	Case 5	Case 6	Case 7	Case 8	Case 9	Case 10
Authors	Tsuboi et al. 2025	Chen et al. 2023	Chen et al. 2023	Onder et al. 2023	Ravat et al. 2022	Magrinelli et al. 2021	Yamashita et al. 2017	Wirth et al. 2017	Choi et al. 2016	Choi et al. 2016	Cif et al. 2014
Sex/current age (y)	M/32	F/38	F/34	M/27	M/20	F/43	M/53	M/25	M/39	M/41	F/15
Country	Japan	China	China	Turkey	India	United Kingdom	Japan	France	South Korea	South Korea	France
Parental consanguinity	No	NA	NA	Yes	No	No	Yes	No	NA	NA	Yes
Family history	PD (Paternal grandmother)	Unremarkable	Unremarkable	Unremarkable	Unremarkable	Unremarkable	Sister affected	NA	Brother affected (case 9)	Brother affected (case 8)	NA
Age at onset (y)	27	33	30	24	17	27	39	23	29	35	3
Symptoms at onset	Dystonia	Bradykinesia	Bradykinesia, stiffness	Bradykinesia, walking difficulty	Bradykinesia, stiffness, resting tremor	Dystonia	NA	Bradykinesia and hypertonia	NA	NA	Social and communication difficulties
Motor features	Parkinsonism, wearing off, dyskinesia, dystonia	Parkinsonism, wearing off, dyskinesia, postural instability	Parkinsonism, wearing off, dyskinesia, dystonia, ataxic gait, postural instability	Parkinsonism,wearing off, dystonia, pyramidal signs	Parkinsonism,wearing off, dyskinesia, ataxic gait	Parkinsonism, dyskinesia, dystonia, pyramidal signs, cerebellar signs, myoclonus, postural instability	Parkinsonism, wearing off, dyskinesia, dystonia, apraxia of eye-opening, hyperreflexia, postural instability	Parkinsonism, dystonia, wearing off, dyskinesia	Parkinsonism, wearing off, dyskinesia	Parkinsonism, wearing off, dyskinesia	Generalized dystonia, spasticity, myoclonus, cerebellar signs, tremor, oculogyric crisis, ocular dysmetria
Nonmotor features	Depression, anxiety, sleep disturbance, panic disorder, pain, urinary frequency, hyperhidrosis	Cognitive impairment, depression, anxiety, fatigue, pain, Sleep disturbance, hyperhidrosis, constipation, urinary symptoms	Sleep disturbance, RBD, decreased appetite, anxiety, depression	Constipation,RBD, cognitive impairment, optic atrophy	Cognitive impairment	Cognitive impairment, anxiety, depression	Incontinence, urinary urgency, hallucination, psychosis, olfactory disorder, restless Leg	Depression, anxiety, aggressiveness, apathy, ICB, DDS, hallucination, delusions	NA	NA	Developmental cognitive impairment, atypical absence seizure
Brain MRI	Unremarkable	Mild cerebellar atrophyNo iron deposition	Mild cerebellar atrophyNo iron deposition	Iron accumulation in basal ganglia	Mild cerebellar atrophy	Mild cerebellar atrophyNo iron deposition	Unremarkable	Unremarkable	Unremarkable	Unremarkable	Subtle cerebellar atrophyiron accumulation in globus pallidus and substantia nigra
Response to Levodopa	Good	Good	Good	Good	Good	Good	NA	Good	Good	Good	NA
Disease duration before DBS	3 years	5 years	4 years	3 years	4 years	11 years	NA	2 years	NA	NA	12 years
Duration of medication treatment before DBS	2 years	NA	NA	NA	1 year	1 year	NA	NA	a few years	a few years	NA
DBS target	GPi	STN	STN	STN	STN	GPi	STN	STN	STN	STN	GPi
Response to DBS	Marked improvement in truncal dystonia and dyskinesia with reduced off time. Clinical assessments were summarized in Table 1	Improved motor scores (off-state MDS-UPDRS III, 71 to 35), dyskinesia (MDS-UPDRS IV 4.1–4.2, 2 to 0), non-motor symptoms (MDS-UPDRS I, 17 to 10) at 2-year follow-up	Improved motor scores (off-state MDS-UPDRS III, 56 to 27), dyskinesia (MDS-UPDRS IV 4.1–4.2, 3 to 2), non-motor symptoms (MDS-UPDRS I, 17 to 8) at 4-month follow-up	Almost independent ADL performance with improved motor scores (off-state MDS-UPDRS III, 80 to 35; on-state, 30 to 25) at 8-month follow-up	Excellent dyskinesia relief (>90 %) and OFF-period reduction with improved motor scores (off-state UPDRS III, 61 to 16) at 3-month follow-up	Truncal dystonia improvement with complete dyskinesia cessation, enabling increased levodopa dosage	NA	Marked improvement in motor and non-motor symptoms at 1-year follow-up	Excellent outcome in motor fluctuations	Excellent outcome in motor fluctuations	No recurrence of status dystonicus, near-complete resolution of oculogyric crises, and significant upper-limb tremor reduction with improved object handling at 9-month follow-up
Mutations in *PLA2G6* genes	Compound heterozygous mutations: c.1977C > G (p.N659K); c.2129G > A (p.R710H)	Compound heterozygous mutations: c.1937C > A (p.P646H); c.1A > G (p.M1V)	Compound heterozygous mutations: c.991G > T (p.D331Y); c.1934_1938del (p.R645Qfs*?)	Homozygous mutation: c.1894C > T (p.R632W)	Homozygous mutation: c.2222G > A (p.R741Q)	Compound heterozygous mutations: c.956C > T (p.T319M); c.1061 T > C (p.L345P)	homozygous mutation c.1495G > A (p.A499T)	Compound heterozygous mutations: c.109C > T (p.R37X); c.2321G > T (p.S774I)	Compound heterozygous mutations: c.359G > A (p.W120*); c.1742G > A (p.R581Q)	Compound heterozygous mutations: c.359G > A (p.W120*); c.1742G > A (p.R581Q)	Homozygous mutation: c.1640A > G (p.E547G)

Abbreviations: DBS, deep brain stimulation; GPi, globus pallidus internus; MDS UPDRS, Movement Disorder Society Unified Parkinson’s Disease Rating Scale; NA, not available; PD, Parkinson's disease; RBD, REM sleep behavior disorder; STN, subthalamic nucleus.

The review revealed that DBS was typically performed within five years of symptom onset, in response to the atypically rapid emergence of motor complications in PARK14 compared to common PD phenotypes. Eight patients received STN DBS and two received GPi DBS, including the present case. Favorable motor outcomes were reported across both targets, with improvements noted in OFF-period parkinsonism, dyskinesia, dystonia, and functional independence. Notably, dystonia, a prominent feature in our patient, responded well to GPi DBS [2]. However, the existing literature has limitations; few studies used quantitative motor scales like the MDS-UPDRS, and systematic reporting on non-motor symptoms and patient-reported outcomes was scarce. Follow-up durations ranged from 3 months to 3 years, with our case representing the longest postoperative follow-up to date.

Our case contributes significantly to this small body of evidence by providing the longest postoperative follow-up to date, with systematic documentation of motor, non-motor, and quality-of-life outcomes. The sustained improvement in motor complications and quality of life over three years confirms the long-term efficacy of GPi DBS for PARK14. Concurrently, the observed cognitive decline underscores that DBS, while effective for motor symptoms, does not alter the underlying neurodegenerative course. Furthermore, the patient's history of a suicide attempt emphasizes the critical need for comprehensive neuropsychiatric assessment, and in such cases, GPi may be the preferred DBS target [10].

In conclusion, this case report and literature review indicate that DBS is a highly effective therapy for managing the complex motor symptoms of PARK14, potentially improving patient quality of life. As attention shifts towards patient-centered care, future research must incorporate long-term follow-up and comprehensive outcome measures to refine therapeutic strategies for this rare and complex disorder.

## Declaration of competing interest

The authors declare that they have no known competing financial interests or personal relationships that could have appeared to influence the work reported in this paper.

## Appendix A Supplementary data

Supplementary Data 1

Supplementary video 2 Prior to treatment, the patient displayed stereotypical rightward trunk deviation during walking, consistently with each left foot strike. This deviation was absent during long-stride or backward walking, consistent with truncal dystonia. Following deep brain stimulation, the dystonia improved.
