# Supplementary material for: Long-term efficacy of deep brain stimulation in PLA2G6-related Parkinson’s disease: A case report with literature review
Source: Clin Park Relat Disord. 2025 Jul 28;13:100377. doi: 10.1016/j.prdoa.2025.100377 (PMC12337871; doi:10.1016/j.prdoa.2025.100377)
Supplement: Supplementary Data 1 [file mmc1.docx]

**Supplemental Materials for Tsuboi et al. “Long-term Efficacy of Deep Brain Stimulation in *PLA2G6*-Related Juvenile Parkinson’s Disease: A Case Report with Literature Review”**

Contents

- Supplementary Figure 1
- Supplementary Table 1
- Supplementary Methods

**
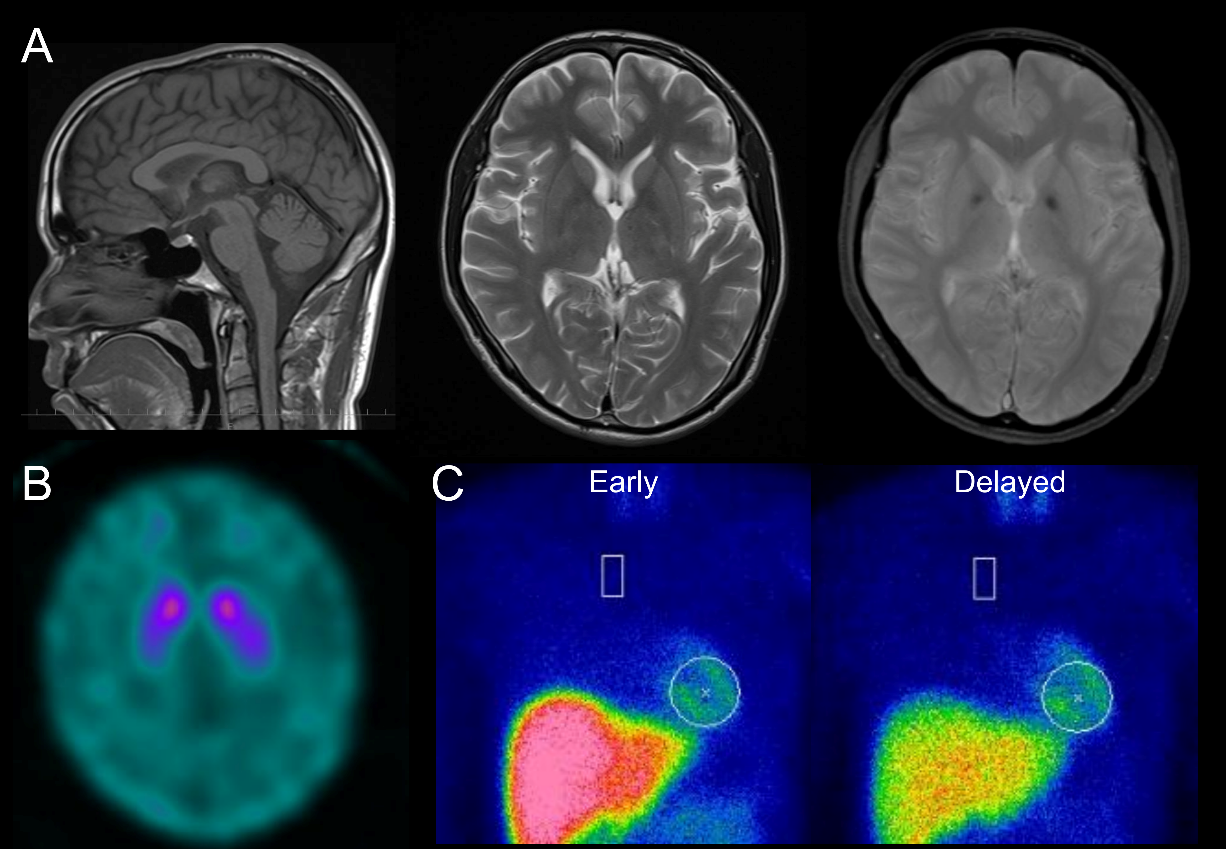
**

**Supplementary Figure 1.** Neuroimaging of the patient. (A) Brain MRI shows normal findings. From left to right: sagittal T1-weighted image, axial T2-weighted image, and axial T2 STIR image. (B) Dopamine transporter single-photon emission computed tomography (DaT SPECT) demonstrates reduced tracer uptake in the bilateral striatum. (C) ^123^I-metaiodobenzylguanidine (MIBG) myocardial scintigraphy reveals normal myocardial uptake both in early and delayed phases.

| Supplementary Table 1. Summary of *PLA2G6* variants identified in the patient | | | | | | | | | | | | | | | |
| --- | --- | --- | --- | --- | --- | --- | --- | --- | --- | --- | --- | --- | --- | --- | --- |
| Nucleotide change | Amino acid change | Exon | Region (GRCh38) | dbSNP | Alelle frequency | | |  | Prediction analysis | | | | | | |
|  |  |  |  |  | jMorp  (ToMMo 60KJPN) | gnomAD v4.1.0 (East Asia) | gnomAD v4.1.0 (Total) |  | CADD | SIFT | PolyPhen2 (HumVar) | Mutation Taster 2021 | Alpha Missense | REVEL | ClinVar |
| c.1977C>G | p.N659K | 14 | chr22:38115584G>T | rs201727354 | 0.000008  (1/119880) | 0 (0/44868) | 0.000001859 (3/1613420) |  | 25.2 | Damaging (0) | Probably damaging  (1) | Deleterious | likely_ pathogenic (0.999) | Disease-causing (0.84) | NA |
| c.2129G>A | p.R710H | 15 | chr22:38113560C>T | rs147455037 | 0.000083 (10/119880) | 0.00004455 (2/44894) | 0.0001091 (176/1613824) |  | 33 | Damaging (0) | Probably damaging  (0.991) | Deleterious | ambiguous (0.508) | Disease-causing (0.754) | Conflicting interpretations of pathogenicity |
| Abbreviations: CADD, Combined Annotation Dependent Depletion; dbSNP, Database of Single Nucleotide Polymorphisms; gnomAD, Genome Aggregation Database; jMorp, Japanese Multi Omics Reference panel; PolyPhen2, Polymorphism Phenotyping v2; SIFT, Sorting Intolerant From Tolerant | | | | | | | | | | | | | | | |

**Supplementary Methods**

We conducted a systematic literature search using Medline (PubMed), covering the period from January 2000 through April 2025. The search terms were: (((PLA2G6) OR (PARK14) OR (NBIA2*) OR (PLAN) OR (PLA2G6-associated neurodegeneration)) AND ((DBS) OR (deep brain stimulation))) AND (English[Language]). The inclusion criteria were: (1) clinical studies, including case series or case reports; (2) involving PLAN patients treated with DBS; and (3) written in English.
